# Combined Use of *S. pombe* and *L. thermotolerans* in Winemaking. Beneficial Effects Determined through the Study of Wines’ Analytical Characteristics

**DOI:** 10.3390/molecules21121744

**Published:** 2016-12-18

**Authors:** Ángel Benito, Fernando Calderón, Santiago Benito

**Affiliations:** Chemistry and Food Technology Department, Polytechnic University of Madrid, Avenida Complutense S/N, 28040 Madrid, Spain; angel@urbinavinos.com (A.B.); fernando.calderon@upm.es (F.C.)

**Keywords:** *Schizosaccharomyces pombe*, *Lachancea thermotolerans*, pyruvic acid, malic acid, lactic acid, urea, food safety, amino acids, winemaking

## Abstract

The most common way to produce red wine is through the use of *Saccharomyces cerevisiae* strains for alcoholic fermentation and lactic acid bacteria for malolactic fermentation. This traditional winemaking methodology produces microbiologically stable red wines. However, under specific conditions off-flavours can occur, wine quality can suffer and human health problems are possible, especially after the second fermentation by the lactic acid bacteria. In warm countries, problems during the malolactic fermentation arise because of the high pH of the must, which makes it very difficult to properly control the process. Under such conditions, wines with high acetic acid and histamine concentrations are commonly produced. This study investigates a recent red wine-making technology that uses a combination of *Lachancea thermotolerans* and *Schizosaccharomyces pombe* as an alternative to the conventional malolactic fermentation. This work studies new parameters such as aroma compounds, amino acids, ethanol index and sensory evaluation. *Schizosaccharomyces pombe* totally consumes malic acid while *Lachancea thermotolerans* produces lactic acid, avoiding excessive deacidification of musts with low acidity in warm viticulture areas. This methodology also reduces the malolactic fermentation hazards in wines with low acidity. The main products are wines that contain less acetic acid, less biogenic amines and precursors and less ethyl carbamate precursors than the traditional wines produced via conventional fermentation techniques.

## 1. Introduction

It is currently assumed that regular alcoholic fermentation and malolactic fermentation is the only way to microbiologically stabilize a red wine before bottling. However, Pasteur considered malolactic fermentation as a wine problem during the first studies of wine microbiology because he considered lactic acid bacteria to be wine spoilage microorganisms. Many research groups are now paying attention to the oenological applications of non-*Saccharomyces* yeast strains to improve wine quality [1,2,3,4,5,6]. Some of the most studied non-*Saccharomyces* yeast species in winemaking are *Torulaspora delbrueckii* [7,8], *Kloeckera apiculata* [9], *Hanseniaspora uvarum* [10], *Hanseniaspora vineae* [11], *Candida zemplinina* [12], *Candida pulcherrima* [13], *Schizosaccharomyces pombe* [14], *Hansenula anomala* [15] and *Lachancea thermotolerans* [16,17]. Most of these studies report that sequential inoculation of a non-*Saccharomyces* and a *Saccharomyces cerevisiae* strain is the best option.

*S. pombe* has traditionally been used for deacidification due to its ability to convert the harsh tasting l-malic acid into ethanol, making very acidic wines smoother [18,19,20]. However, organisms of the genus *Schizosaccharomyce*s are also used in winemaking for many other purposes. One new application exploits their ability for higher polysaccharide release during fermentation and ageing over lees [4,21,22]. Another one is their use to decrease gluconic acid concentration in wines from rotten grapes [23,24,25,26,27], as well as color improvement of red wines through their ability for enhanced formation of highly stable pigments such as the vitisins and pyranoanthocyanin [28,29,30]. Finally, from a food safety viewpoint, the genus *Schizosaccharomyces* is being used to produce safer wines [31] because it possess urease activity [32] that avoids ethyl carbamate production and reduces the risk of biogenic amine formation by wild-type lactic acid bacteria [33]. Conversely, *Lachancea thermotolerans* is used in warm regions to produce more acidic wines, with less volatile acidity and higher aroma complexity from low acidic musts [33,34,35,36].

The genus *S. pombe* has not been traditionally used for winemaking due to the occurrence of off-flavours caused by substances such as acetic acid, acetaldehyde, acetoin and ethyl acetate [37,38,39,40,41], which are currently associated with non-selected strains [42]. Recent studies have proven that it is possible to select strains that are appropriate for winemaking [14]. The main problem with such selection processes was the difficulty of isolating a representative number of strains from environmental samples [43], which has caused difficulty until now to obtain collections of representative strains of this genus [44]. Nevertheless, the number of available strains is limited and further selection processes similar to those performed for *S. cerevisiae* for winemaking are required.

This new study combined strains of *L. thermotolerans* and *S. pombe* to make wine from a high pH and high potential alcohol must without using *Saccharomyces* and lactic acid bacteria, to avoid possible collateral effects from malolactic fermentation. Several parameters such as volatile compounds, amino acids, ethanol index and sensory properties of the wines produced with this new biotechnology were investigated for the first time. Additionally, as in previous studies, acid content, pH, glycerol, urea, alcohol and biogenic amines were also determined.

## 2. Results and Discussion

### 2.1. Yeast Population Kinetics

**Figure 1:**
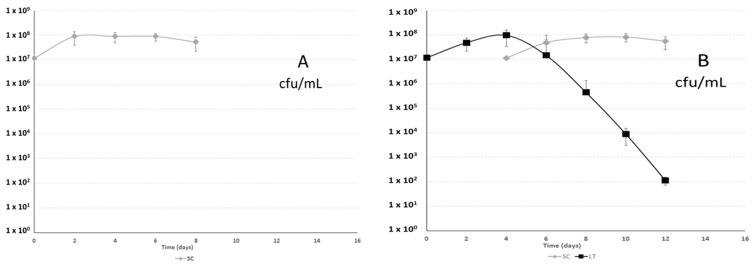

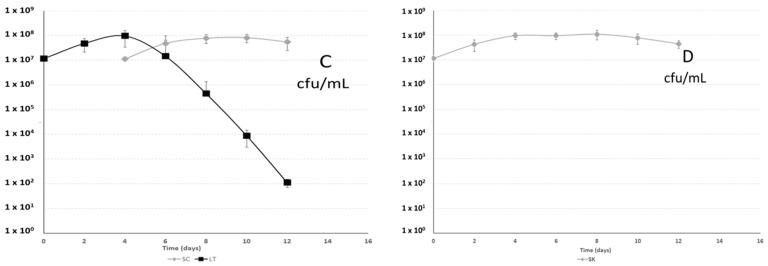
Population development during fermentation of *Saccharomyces cerevisiae* 88 alone (SC; **A**), sequential fermentation with *Saccharomyces cerevisiae* 88 and *Lachancea thermotolerans* CONCERTO™ (LT…SC; **B**), sequential fermentation with *Schizosaccharomyces pombe* 4.5 and *Lachancea thermotolerans* CONCERTO™ (LT…SK; **C**) and *Schizosaccharomyces pombe* 4.5 alone (SK; **D**).

Figure 1 shows the growth of the different yeast strains during fermentation. In sequential fermentations, inoculated with *Saccharomyces cerevisiae* 88 or *Schizosaccharomyces pombe* 4.5, *Lachancea thermotolerans* CONCERTO™ started to decline just after the second inoculation, although the *L. thermotolerans* population decrease was more rapid in the presence of *S. cerevisiae*. The progressive disappearance of *L. thermotolerans* could be explained as a result of the presence of another more well-adapted yeast competitor (*S. cerevisiae* or *S. pombe*) and an ethanol concentration higher than 9% *v*/*v* by day 6. *L. thermotolerans* has been reported to only tolerate up to 9% *v*/*v* ethanol when in a pure culture fermentation [33,34]. This limited alcohol tolerance of *L. thermotolerans* causes difficulty in the production of a dry red wine in warm regions alone without using a yeast with higher ethanol tolerance in a combined fermentation.

### 2.2. Sugar Consumption Kinetics

**Figure 2:**
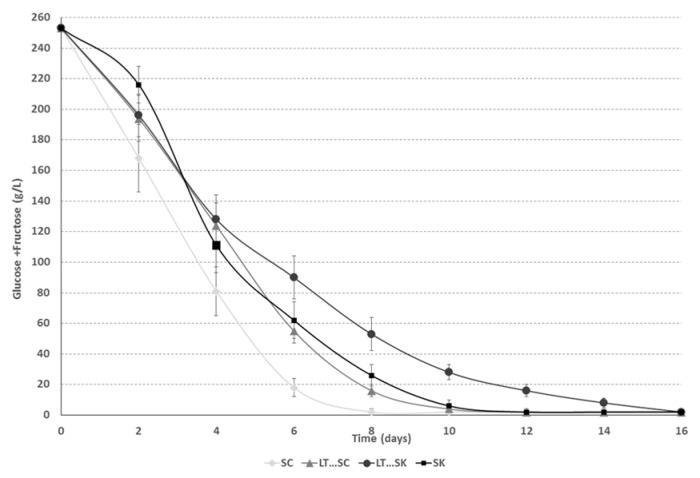
Fermentation kinetics of glucose + fructose for *Saccharomyces cerevisiae* 88 alone (SC), a sequential fermentation with *Saccharomyces cerevisiae* 88 and *Lachancea thermotolerans* CONCERTO™ (LT…SC), a sequential fermentation with *Schizosaccharomyces pombe* 4.5 and *Lachancea thermotolerans* CONCERTO™ (LT…SK) and *Schizosaccharomyces pombe* 4.5 alone (SK).

The consumption kinetics of glucose and fructose were more rapid when *S. cerevisiae* strain 88 was involved (Figure 2) than when *L. thermotolerans* and *S. pombe* were used. The alcoholic fermentation times varied from 8 to 16 days. All alcoholic fermentations finished correctly, reaching concentrations lower than 2 g/L of glucose and fructose (Figure 2 and Table 1). Other authors have previously described slower fermentation kinetics for *L. thermotolerans* [16,17] and *S. pombe* [38] than for *S. cerevisiae*. Musts with high sugar contents have been reported to be improperly fermented by *L. thermotolerans* alone [34].

### 2.3. Acetic Acid

The maximal final concentration of acetic acid was 0.49 g/L for a malolactic fermentation following an alcoholic fermentation by *S. cerevisiae* in pure culture (Table 1). Alcoholic fermentations alone did not show significant differences, with values of approximately 0.37 g/L. Previous studies reported the *L. thermotolerans* produced less acetic acid than *S. cerevisiae* [36,45]. The genus *Schizosaccharomyces* has been previously reported as producing more acetic acid than *S. cerevisiae,* with acetic acid concentrations up to 1 g/L [29]. However, some *S. pombe* strains have been recently selected for their low acetic acid production [14,42], and the results for those strains agree with the results obtained in this study.

### 2.4. Malic Acid

**Figure 3:**
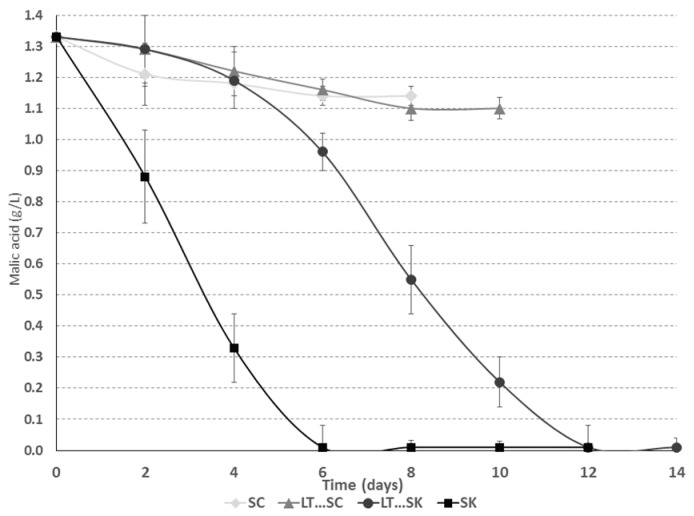
Fermentation kinetics of L-malic acid for *Saccharomyces cerevisiae* 88 alone (SC), a sequential fermentation with *Saccharomyces cerevisiae* 88 and *Lachancea thermotolerans* CONCERTO™ (LT…SC), a sequential fermentation with *Schizosaccharomyces pombe* 4.5 and *Lachancea thermotolerans* CONCERTO™ (LT…SK) and *Schizosaccharomyces pombe* 4.5 alone (SK).

Malic acid was completely degraded in all trials with *S. pombe* (Figure 3 and Table 1) during alcoholic fermentation. The *S. cerevisiae* strain degraded 14% of the initial malic acid content in the must (Figure 3 and Table 1).

Several authors have reported similarly high malic acid degradations for yeast in genera other than *Schizosaccharomyces*, which varied from 10 to 20% [7,42] or even up to 39% for specific hybrids [46], but no one has reported the total degradation of malic acid (i.e., 100%) by those genera. The malic acid reduction clearly affected the final pH value of the fermentations (Table 1) because *S. pombe* fermentations had a final pH greater than 4. *O. oeni* metabolized malic acid to lactic acid in a malolactic fermentation (Table 1).

### 2.5. L-Lactic Acid

**Figure 4:**
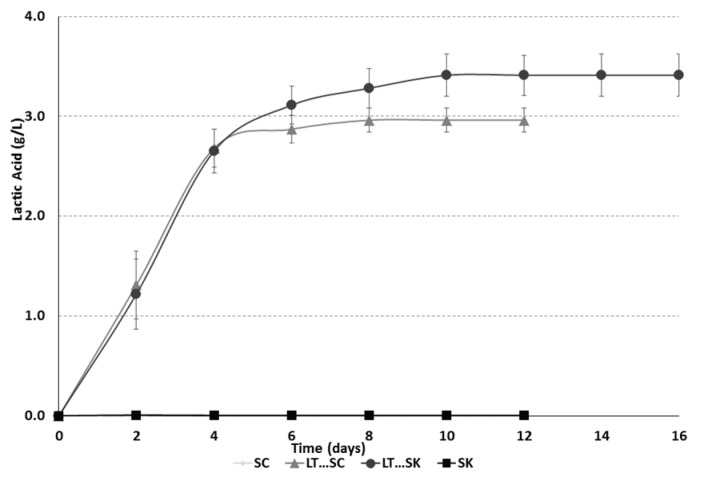
Fermentation kinetics of L-lactic acid for *Saccharomyces cerevisiae* 88 alone (SC), a sequential fermentation with *Saccharomyces cerevisiae* 88 and *Lachancea thermotolerans* CONCERTO™ (LT…SC), a sequential fermentation with *Schizosaccharomyces pombe* 4.5 and *Lachancea thermotolerans* CONCERTO™ (LT…SK) and *Schizosaccharomyces pombe* 4.5 alone (SK).

Fermentations involving *L. thermotolerans* produced l-lactic acid during alcoholic fermentation (Figure 4; Table 1). The final concentration of L-lactic acid produced by *L. thermotolerans* in this study varied from 2.96 to 3.41 g/L (Table 1), which reduced the final pH (Table 1). Previous studies have reported significant acidification from l-lactic acid, varying from 0.22 g/L to 6.38 g/L when mixed cultures of *Lachancea thermotolerans* were used with the main objective of increasing the acidity of the must [17,33,36]. Experiments involving malolactic fermentations showed an increase in l-lactic acid of approximately 0.73 g/L (Table 1). These final l-lactic acid concentration levels were significantly lower than the ones obtained using *L. thermotolerans* for the studied must. This phenomenon could be explained in that the initial level of malic acid of 1.33 g/L was low compared to northern viticulture regions that produce a must with higher initial malic acid concentration.

### 2.6. Pyruvic Acid

**Figure 5:**
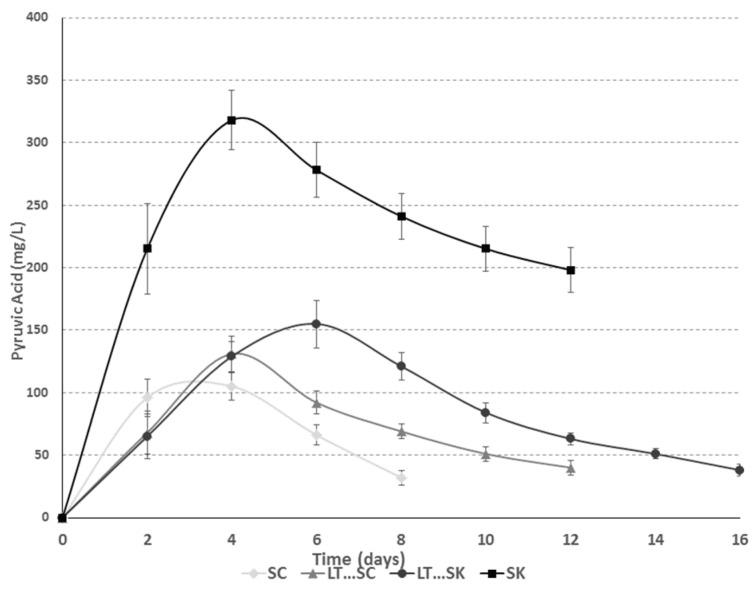
Fermentation kinetics of pyruvic acid for *Saccharomyces cerevisiae* 88 alone (SC), sequential fermentation with *Saccharomyces cerevisiae* 88 and *Lachancea thermotolerans* CONCERTO™ (LT…SC), a sequential fermentation with *Schizosaccharomyces pombe* 4.5 and *Lachancea thermotolerans* CONCERTO™ (LT…SK) and *Schizosaccharomyces pombe* 4.5 alone (SK).

All fermentations involving *S. pombe* produced a higher pyruvic acid concentration than the others (Figure 5). The maximum values were obtained during the first days of alcoholic fermentation (Figure 5), except for sequential fermentation with *L. thermotolerans* and *S. pombe,* in which the maximum concentration was reached at day 8. A pure culture of *S. pombe* produced a maximum pyruvic acid concentration of 318 mg/L after 96 h of fermentation. Other authors have reported higher values of up to 487 mg/L for some selected *S.pombe* strains in pure culture [42]. Specific *S. cerevisiae* strains have also been reported to produce a maximum pyruvic acid concentration as high as 150 mg/L [47], which is higher than for *S. cerevisiae* strain 88. Greater pyruvic acid formation could be related to the higher colour intensity observed in this study for *S. pombe* fermentations because this compound is related to the formation of highly stable coloured pigments such as vitisin A [17,30,48].

### 2.7. Glycerol

The genera *Schizosaccharomyces* and *Lachancea* have been described as higher glycerol producers than the genus *Saccharomyces* [17,36,45]. The final levels of glycerol varied from 8.88 g/L to 9.66 g/L (Table 1). *S. pombe* produced the highest concentration (Table 1). A high glycerol content has been described as one of the main contributions of non-*Saccharomyces* strains to wine quality [1,49]. Nevertheless, other authors have reported that species such as *Candida stellata* could effectively produce high concentrations of glycerol of up to 14 g/L [1].

### 2.8. Ethanol

The ethanol levels varied from 14.22 to 14.78 (% *v*/*v*) (Table 1). Other authors have reported that *S. pombe* is highly resistant to ethanol stress conditions [50]. Sugar metabolism can be used to synthetize compounds other than ethanol, such as glycerol or pyruvic acid, or to increase the biomass of the yeast [51,52]. The results show that fermentations involving *L. thermotolerans* and *S. pombe* produced lower ethanol levels than *S. cerevisiae*. These data are in accord with other authors who confirmed that some non-*Saccharomyces* yeasts produced lower ethanol yields than *Saccharomyces* [27,53,54,55,56]. Previous studies have shown similar results for *L. thermotolerans* [36] and *S. pombe* [53]. Nevertheless, the differences (Table 1) were approximately 0.56% (*v*/*v*). Some authors have recently reported more significant ethanol reductions greater than 1% (*v*/*v*) using non-*Saccharomyces* strains, which may be related to specific conditions of high aeration [57,58] or via the use of glucose oxidase and catalase [59].

### 2.9. Urea

The final concentration of urea in the completed alcoholic fermentations was lower in fermentations involving *S. pombe*, with values less than 0.2 mg/L (Table 1). This effect was attributed to the enzymatic capacity of *Schizosaccharomyces* to produce urease [32,60], the activity of which has been proposed as a means of reducing the hazard of ethyl carbamate formation (one of the most toxic compounds reported in wine) [14,31] in winemaking because urease eliminates urea, the main precursor of ethyl carbamate. This factor is becoming increasingly important because ethyl carbamate is a known carcinogen that is present in a variety of fermented foods [61]. Some countries such as the USA, Japan and Canada have established legal limits.

### 2.10. Citric Acid

No statistical differences in citric acid were observed during any alcoholic fermentation (Table 1). However, in an experiment in which *O. oeni* was inoculated after an alcoholic fermentation, most of the citric acid was consumed (Table 1). An increase in the acetic acid concentration was also detected during the same period, so citric acid could have been converted into acetic acid by the lactic acid bacteria; such a collateral effect usually increases the final acetic acid concentration [29,62] and decreases the wine quality.

### 2.11. Volatile Aromatics

The concentrations of higher alcohols were higher in fermentations involving *L. thermotolerans* and *S. cerevisiae* than in those involving *S. pombe* (Table 2). Several non-*Saccharomyces* yeast species produced less of the higher alcohols than *S. cerevisiae* [7,17,36,47,63,64,65], but great variability among strains has been reported [10]. *S. pombe* has been reported to produce more of the higher alcohols than *S. cerevisiae* [30], but selected strains have also been described as low producers [14].

The production of wines with lower levels of higher alcohols has been reported as a way to produce wines with typicity for specific grape varieties or to increase wine complexity [66]. Similarly, fermentation with *S. pombe* alone produced a lower concentration of esters such as isoamyl acetate. Compounds considered negative for winemaking, such as ethyl acetate and diacetyl, were higher in fermentations involving malolactic fermentation. Similar results have been reported previously [29,67]. Acetaldehyde levels were reduced during trials involving malolactic fermentation (Table 2). Ethyl lactate was higher in fermentations involving *L. thermotolerans* or malolactic fermentation.

### 2.12. Biogenic Amines

Biogenic amines [68,69,70,71] have been proven to be harmful for human health, so they must be taken into account for food safety. A histamine concentration of 2 mg/L is the highest allowable concentration in some countries [72]. The final levels of histamine in all fermentations were lower than 2 mg/L (Table 3). Fermentations involving *S. pombe* showed lower concentrations than those involving a malolactic fermentation (Table 3). *Schizosaccharomyces* has been reported as effective in reducing the risk of the formation of biogenic amines [20,29,33] or ethyl carbamate precursors [14,31].

No significant differences were observed for any biogenic amine, except for histamine when a malolactic fermentation occurred (Table 3). However, the histamine levels were always below 2 mg/L. Other authors have reported slight differences related to the ability of yeast strains to remove biogenic amines during fermentation [14,29,73]. This phenomenon was not observed in this study, probably due to the low initial level of biogenic amines in the initial must (Table 3). Biogenic amine levels mainly increase during wine ageing and malolactic fermentation [74,75,76,77].

### 2.13. Amino Acids

Higher final concentrations of most amino acids occurred in *S. pombe* fermentations (Table 4). Previous studies reported *S. pombe* as demanding less nitrogen [30] and releasing more nitrogen than *S. cerevisiae* [14]. *S. cerevisiae* and *L. thermotolerans* fermentations produced a higher final concentration of ornithine than *S. pombe* (Table 4). Some authors observed a relationship between threonine, valine, isoleucine and leucine (Table 4) and the higher alcohols 1-propanol, isobutanol, 2-methylbutanol and 3-methylbutanol [45], which explains the differences observed in the final concentrations of higher alcohols after alcoholic fermentation in the presence of these amino acids (Table 4) because they are precursors of higher alcohols (Table 4) [45]. *S. pombe* fermentations had higher concentrations in the amino acid precursors of higher alcohols (Table 4). *S. pombe* pure fermentations showed increased histidine, tyrosine and lysine concentrations; those amino acids are biogenic amine precursors [72,74]. This phenomena was not observed for a combined fermentation with *S. pombe* and *L. thermotolerans*. The transformation of some precursors into biogenic amines occurs during a malolactic fermentation [74,75,76]; therefore, the wines fermented by *S. pombe* did not have a serious risk of high levels of histamine or tyramine because a long malolactic fermentation was unnecessary [31]. On the other hand, combined fermentations with *L. thermotolerans* and *S. pombe* were characterized by lower levels of biogenic amine precursors than *S. pombe* alone, even though malolactic fermentation was also not required (Table 4). Therefore, such biotechnology could be important for wine ageing or ageing over lees because such wines would be more acidic and have a lower content of biogenic amine precursors than wines fermented by *S. pombe* alone. The wines involving a malolactic fermentation showed a reduction in arginine and histidine, and a slight increase in glycine, alanine and ornithine.

### 2.14. Colour Measurements

Table 5 shows the results of colour assessments for the different treatments. Fermentations involving *S. pombe* alone showed higher colour intensity values. Similar results have been reported previously [29]. Significant differences were observed in the hue parameters when malolactic fermentation took place.

### 2.15. Ethanol Index

**Figure 6:**
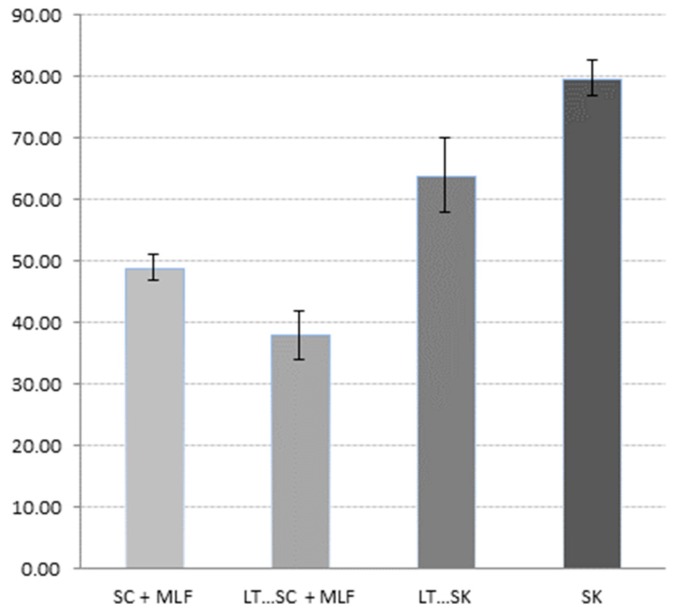
Results of the ethanol index analysis of bottled wines from different fermentation processes of *Saccharomyces cerevisiae* 88 alone (SC), sequential fermentation with *Saccharomyces cerevisiae* 88 and *Lachancea thermotolerans* CONCERTO™ (LT…SC), sequential fermentation with *Schizosaccharomyces pombe* 4.5 and *Lachancea thermotolerans* CONCERTO™ (LT…SK), *Schizosaccharomyces pombe* 4.5 alone (SK), and fermentations after malolactic fermentation with *Oenococcus oeni* 217 (+ MLF).

Some authors have described some non-*Saccharomyces* and specifically *Schizosaccharomyces* yeasts as higher producers of polysaccharides [4,22,78]. To estimate the content of polysaccharides, the ethanol index was used in this study (Figure 6). Fermentations involving *S. pombe* showed higher ethanol index values. These results match the information gave by previous authors [4,22].

### 2.16. Sensory Evaluation

**Figure 7:**
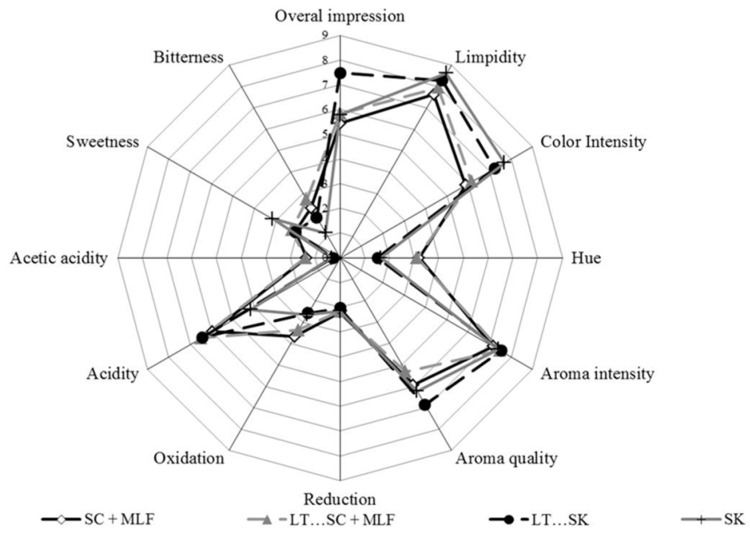
Results of the sensory analysis of bottled wines from different fermentation processes of *Saccharomyces cerevisiae* 88 alone (SC), sequential fermentation with *Saccharomyces cerevisiae* 88 and *Lachancea thermotolerans* CONCERTO™ (LT…SC), sequential fermentation with *Schizosaccharomyces pombe* 4.5 and *Lachancea thermotolerans* CONCERTO™ (LT…SK), *Schizosaccharomyces pombe* 4.5 alone (SK), and fermentations after malolactic fermentation with *Oenococcus oeni* 217 (+ MLF). The intensity of the 12 attributes was scaled from 0 (no character) to 10 (very strong character).

Figure 7 shows the spider web diagram of the average scores of the taste and olfactory attributes that were assessed. Large differences in the perception of acidity were recorded; this result agrees with the acidity parameters explained above (Table 1). Alcoholic fermentation followed by malolactic fermentation produced a slightly stronger sensation of oxidation and acetic acidity. Nevertheless, no serious faults were reported for any of the wines. None of the wines produced by fermentation with *S. pombe* showed any perceptible organoleptic problems; indeed, the combination between *S. pombe* and *L. Thermotolerans* received the best scores from all tasters. Although all fermentations involving *S. pombe* achieved the main goals related to microbiological malic acid stabilization. The preferred fermentation strategy was a combined use of *S. pombe* and *L. Thermotolerans*, probably because the fermented must was less standardized, fruitier and possessed a higher acidity. Differences in the colour intensity could be explained by the pyruvic acid production noted above or the different absorption of coloured compounds by the yeast species (Table 5).

## 3. Materials and Methods

### 3.1. Microorganisms

The following yeast strains were used for the experimental fermentations: *Kluyveromyces thermotolerans* Concerto™ (Hansen, Hørsholm, Denmark; www.chr-hansen.com) that belongs to the yeast species *Lachancea thermotolerans*, *Saccharomyces cerevisiae* 88 (Spanish Type Culture Collection, Valencia, Spain) and *Schizosaccharomyces pombe* 4.5 (Chemistry and Food Technology department, Polytechnic University of Madrid, Madrid, Spain [42]. The strain of lactic acid bacteria used was *Oenococcus oeni* 217 (Spanish Type Culture Collection).

### 3.2. Vinification

All fermentations used a must of *Vitis vinifera* L. cultivar Tempranillo grapes grown at the El Socorro Experimental vineyard (Madrid, Spain). The must was pasteurized at 105 °C for 5 min. A microvinification method similar to that described in the scientific literature was used [45]. Pasteurized must (4 L) was placed in a 5 L glass tank, allowing adequate space for the release of carbon dioxide during fermentation. No sulphur dioxide was added. The sugar concentration was 253 g/L, pH = 3.88, primary amino nitrogen (PAN) 144 g/L, malic acid 1.33 g/L, citric acid 0.2 g/L, lactic and acetic acid bellow 0.1 g/L. To provide nutrition 40 g/hL of Actimax NATURA (Agrovín S.A., Ciudad Real, Spain) were added. Four treatments were used (all in triplicate): (i) inoculation of the must with *S. cerevisiae* 88 (10^7^ CFU/mL) alone (SC); (ii) inoculation of the must with *L. thermotolerans* Concerto™ (10^7^ CFU/mL) followed by *S. cerevisiae* 88 (10^7^ CFU/mL) 96 h later (LT…SC); (iii) inoculation of the must with *L. thermotolerans* Concerto™ (10^7^ CFU/mL) followed by *S. pombe* 4.5 (10^7^ CFU/mL) 96 h later (KT…SK); and (iv) inoculation of the must with *S. pombe* 4.5 (10^7^ CFU/mL) alone (SK). Yeasts were inoculated using 400 mL of sterilized must containing 10^8^ CFU/mL (determined using a Thomas chamber). To reach this population, 100 μL of each yeast suspension were cultivated in 10 mL of YEPD at 25 °C for 24 h. This procedure was repeated three times successively before the final inoculation of 4 mL in the inocula. All inoculations were performed in 500-mL flasks sealed with a Müller valve filled with 98% H_2_SO_4_ (Panreac, Barcelona, Spain), which allowed the release of CO_2_ while avoiding microbial contamination [46]. The temperature was maintained at 25 °C for 72 h before inoculation. The inocula were developed under anaerobic conditions. All fermentations were performed in triplicate. All fermentation processes were carried out at 25 °C. When the sugar content was below 2 g/L, the wines were racked and stabilized for 7 days at 4 °C, after which the final product was bottled. Then, a concentration of 50 mg/L of sulphur dioxide in potassium metabisulphite form was added. Sealed bottles were placed horizontally in a climate chamber at 4 °C until the sensory evaluation took place. The wines fermented with *Saccharomyces cerevisiae* alone (SC) were stabilized and racked following the same procedure when the malolactic fermentation by *Oenococcus oeni* 217 (10^7^ CFU/mL) was finished in 2.8 L vessels at 18 °C. These wines remained under the same storage conditions described above, for one month before the tasting sessions took place.

### 3.3. Measurements of Biochemical Compounds And pH

Determination of glucose + fructose, malic acid, l-lactic acid, acetic acid, pyruvic acid, urea, ethanol index [79] and glycerol concentrations as well as colour intensity (Table 1) were conducted using a Y15 Autoanalyser and a Y350 Semiautomatic Analyzer (Biosystems, Barcelona, Spain). The kits used to perform the analyses were obtained from Biosystems (www.biosystems.es). The quantification metrology was performed according to the manufacturer´s indications from the standards contained in the commercial kits (www.biosystems.es). The alcohol content was determined by using the GAB Microebu ebuillometry method (http://shop.gabsystem.com). The pH was measured with a Crison pH Meter Basic 20 (Crison, Barcelona, Spain).

### 3.4. Microvinification Growth Kinetics

Aliquots were periodically taken aseptically during fermentation and further ten-fold serial dilutions were made. The yeast growth kinetics were monitored by plating 100 μL of the appropriate dilution on lysine media (non-*Saccharomyces* counts; [80]), YEPD media (total yeast counts; [81,82]) and YEPDActBzCl media (*Schizosaccharomyces* counts; [43]) with actidione and benzoic acid as the main inhibitors. In LT…SC fermentations, the population of *Lachancea thermotolerans* was estimated by the difference between the YEPD and the Lysine media counts. In LT…SK fermentations, the population of *Lachancea thermotolerans* was estimated by the difference between the YEPD and YEPDActBzCl media counts. Colonies were counted after growth at 30 °C for 48–72 h. Lactic acid bacteria were monitored in MRS agar (Oxoid, Basingstoke, UK).

### 3.5. Quantification of Volatile Compounds

Volatile compounds (Table 2) were quantified by headspace gas chromatography-mass spectrometry (HS-GC-MS). Analyses were carried out using a Clarus 500 gas chromatograph (Perkin-Elmer, Waltham, MA, USA) equipped with a flame ionization detector coupled to a single quadrupole Clarus 560 S mass spectrometer, all coupled to an Turbomatrix 110 Trap automatic headspace sampler (Perkin-Elmer). The headspace sampler conditions were: thermostat temperature: 80 °C, time of thermostating: 45 min, type of trap: Tenax TA, cycles of purge and trap: 4, temperature of trap capture: 45 °C, desorption temperature of the trap: 290 °C, time of dry trap purge: 10 min, desorption time of trap: 2 min, trap cleaning time: 5 min, needle temperature: 110 °C, temperature of HS-GC transfer line: 150 °C, vial pressure: 30 psi and constant pressure column: 28 psi. A FFAP capillary column (60 m × 0.25 mm DI × 0.25 μm film thickness) was used. Helium (Air Liquide, Paris, France) was used as the carrier gas. A gradient analysis was run using the following temperature program: 40 °C (3 min); 40–80 °C (2 °C/min); 80–180 °C (3 °C/min); and 210 °C (5 min). Identification of individual compounds was based on a comparison of the mass spectra of the individual chromatographic peaks with those valid for the standards and available from the National Institute of Standards and Technology (Gaithersburg, MD, USA) software library. We also compared the retention times for individual peaks from the wine samples with those of the known volatile components to use as standard patterns. We used gas chromatography quality compounds as the sets of the volatile standards for this purpose (Fluka, Sigma-Aldrich Corp., Buchs SG, Standort, Switzerland). The apparatus was calibrated with a 4- meth-yl-2-pentanol internal standard at 50 mg/L. An individual calibration for each volatile compound was accomplished using an external standard at six concentrations ranging from 1 to 500 mg/L. The R2 values were greater than 0.999 for all compounds tested. The detection limit of the method was 0.1 mg/L.

### 3.6. Quantification of Biogenic Amines

Biogenic amines (Table 3) were analysed using a series X-LCTM UHPLC chromatograph (Jasco, Tokyo, Japan), equipped with a 3120-FP fluorescence detector. Gradients of solvent A (methanol/acetonitrile, 50:50, *v*/*v*) and B (sodium acetate/tetrahydrofuran, 99:1, *v*/*v*) were used in a C18 (HALO, Wilmington, DE, USA) column (100 mm × 2.1 mm; particle size 2.7 µm) as follows: 60% B (0.25 mL/min) from 0 to 5 min, 60%–50% B linear (0.25 mL/min) from 5 to 8 min, 50% B from 8 to 9 min, 50%–20% B linear (0.2 mL/min) from 9 to 12 min, 20% B (0.2 mL/min) from 12 to 13 min, 20%–60% B linear (0.2 mL/min) from 13 to 14.5 min, and re-equilibration of the column from 14.5 to 17 min. Detection was performed by scanning in the 340–420 nm range. Quantification was performed by comparison against appropriate external standards. The different amines were identified by their retention times.

### 3.7. Analytical Determination of Amino Acids

Selected amino acids (Table 4) were analysed using a Jasco series X-LCTM UHPLC chromatograph, equipped with a 3120-FP fluorescence detector. Gradients of solvent A (methanol/acetonitrile, 50:50, *v*/*v*) and B (sodium acetate /tetrahydrofuran, 99:1, *v*/*v*) were used in a C18 (HALO) column (100 mm × 2.1 mm; particle size 2.7 µm) as follows: 90% B (0.25 mL/min) from 0 to 6 min, 90%–78% B linear (0.2 mL/min) from 6 to 7.5 min, 78% B from 7.5 to 8 min, 78%–74% B linear (0.2 mL/min) from 8 to 8.5 min, 74% B (0.2 mL/min) from 8.5 to 11 min, 74%–50% B linear (0.2 mL/min) from 11 to 15 min, 50% B (0.2 mL/min) from 15 to 17 min, 50%–20% B linear (0.2 mL/min) from 17 to 21 min, 20%–90% B linear (0.2 mL/min) from 21 to 25 min, and the column was re-equilibrated from 25 to 26 min. Detection was performed by scanning in the 340–455 nm range. Quantification was performed by comparison against appropriate external standards. The different amino acids were identified by their retention times.

### 3.8. Sensory Analysis

The final wines were assessed in a blind test by a panel of 15 experienced wine tasters, all of whom were staff members of the Chemistry and Food Technology Department (Madrid, Spain) and the Estación Enológica de Haro (Haro, Spain). Following the generation of a consistent terminology by consensus, three visual descriptors, four aromas and four taste attributes were chosen to describe the wines. Descriptors such as acidity, acetic acid or aroma were chosen in order to contrast the chemical results obtained in the study. The panellists used a 10 cm unstructured scale, from 0 (no defect) to 10 (very strong perceptible defect) to rate the intensity of the 12 attributes.

### 3.9. Statistical Analyses

All statistical analyses were performed using PC Statgraphics v.5 software (Graphics Software Systems, Rockville, MD, USA). The significance was set to *p* < 0.05 for the ANOVA matrix F value. A multiple range test was used to compare the means.

## 4. Conclusions

A combination of selected *Schizosaccharomyces pombe* and *Lachancea thermotolerans* yeast strains is an alternative to the traditional malolactic fermentation that positively affects the quality of wine produced from musts with low acidity. The results from the fermentation trails showed positive differences in several previous studied parameters such as acetic acid, biogenic amines, glycerol or colour. Fermentations involving *S. pombe* showed volatile profiles with lower concentrations in higher alcohols while fermentations involving malolactic fermentation showed higher levels in ethyl acetate and diacetyl. Fermentations involving *S. pombe* resulted in higher levels in final amino acids. Combined fermentations between *S. pombe* and *L. thermotolerans* reported higher levels of ethanol index that are related to higher polysaccharides releases. Mixed fermentation involving *S. pombe* and *L. thermotolerans* achieved the highest score in overall impression.

## Figures and Tables

**Table 1:** Final analysis of fermentations: *Saccharomyces cerevisiae* 88 alone (SC), sequential fermentation with *Saccharomyces cerevisiae* 88 and *Lachancea thermotolerans* CONCERTO™ (LT…SC), sequential fermentation with *Schizosaccharomyces pombe* 4.5 and *Lachancea thermotolerans* CONCERTO™ (LT…SK), *Schizosaccharomyces pombe* 4.5 alone (SK), and fermentations after a malolactic fermentation with *Oenococcus oeni* 217 (+MLF).

Compound/Property	SC	SC + MLF	LT···SC	LT···SC + MLF	LT···SK	SK
l-Lactic Acid (g/L)	0.01 ± 0.01 _a_	0.73 ± 0.06 _b_	2.96 ± 0.12 _c_	3.71 ± 0.18 _d_	3.41 ± 0.23 _d_	0.02 ± 0.02 _a_
l-Malic Acid (g/L)	1.14 ± 0.03 _b_	0.01 ± 0.01 _a_	1.10 ± 0.05 _b_	0.01 ± 0.01 _a_	0.01 ± 0.01 _a_	0.01 ± 0.01 _a_
Acetic Acid (g/L)	0.38 ± 0.02 _a_	0.49 ± 0.04 _b_	0.35 ± 0.03 _a_	0.43 ± 0.04 _b_	0.35 ± 0.04 _a_	0.39 ± 0.02 _a_
Glucose+Fructose (g/L)	1.55 ± 0.21 _b_	0.09 ± 0.03 _a_	1.76 ± 0.32 _b_	0.14 ± 0.05 _a_	1.98 ± 0.43 _b_	1.72 ± 0.24 _b_
Glycerol (g/L)	8.88 ± 0.02 _a_	8.92 ± 0.04 _a_	9.13 ± 0.05 _b_	9.11 ± 0.08 _b_	9.38 ± 0.06 _c_	9.66 ± 0.02 _d_
Urea (mg/L)	2.62 ± 0.02 _b_	5.18 ± 0.08 _c_	2.58 ± 0.05 _b_	5.23 ± 0.11 _c_	0.14 ± 0.04 _a_	0.12 ± 0.02 _b_
Citric Acid (g/L)	0.18 ± 0.01 _b_	0.02 ± 0.01 _a_	0.17 ± 0.04 _b_	0.04 ± 0.03 _a_	0.18 ± 0.03 _b_	0.19 ± 0.01 _b_
Alcohol (% *v/v*)	14.7 ± 0.0 _d_	14.7 ± 0.0 _d_	14.5 ± 0.0 _c_	14.5 ± 0.0 _c_	14.2 ± 0.0 _a_	14.3 ± 0.0 _b_
pH	3.90 ± 0.02 _c_	3.96 ± 0.03 _d_	3.71 ± 0.04 _a_	3.75 ± 0.03 _a_	3.69 ± 0.04 _a_	4.06 ± 0.02 _d_

Results are the mean ± SD of three replicates. Means in the same row with the same letter are not significantly different (*p* < 0.05).

**Table 2:** Final analysis of volatile compounds from fermentations by *Saccharomyces cerevisiae* 88 alone (SC), sequential fermentation with *Saccharomyces cerevisiae* 88 and *Lachancea thermotolerans* CONCERTO™ (LT···SC), sequential fermentation with *Schizosaccharomyces pombe* 4.5 and *Lachancea thermotolerans* CONCERTO™ (LT…SK), *Schizosaccharomyces pombe* 4.5 alone (SK), and fermentations after malolactic fermentation with *Oenococcus oeni* 217 (+MLF).

Compounds (mg/L)	SC	SC + MLF	LT···SC	LT···SC + MLF	LT···SK	SK
Acetaldehyde	21.56 ± 1.88 _c_	2.58 ± 0.27 _a_	17.83 ± 2.56 _cb_	3.12 ± 0.58 _a_	15.32 ± 2.02 _b_	16.21 ± 1.74 _b_
Ethyl lactate	2.88 ± 0.22 _a_	22.52 ± 1.16 _d_	16.42 ± 0.89 _b_	29.63 ± 2.32 _e_	19.78 ± 1.02 _c_	3.38 ± 0.31 _a_
Ethyl acetate	19.42 ± 2.15 _a_	32.42 ± 2.83 _b_	21.58 ± 3.01 _a_	30.62 ± 3.88 _b_	19.83 ± 2.74 _a_	18.77 ± 2.32 _a_
Diacetyl	2.22 ± 0.18 _a_	13.46 ± 1.08 _b_	2.41 ± 0.37 _a_	11.64 ± 2.36 _b_	2.35 ± 0.41 _a_	2.16 ± 0.21 _a_
Isoamyl acetate	3.73 ± 0.45 _b_	3.46 ± 0.41 _b_	3.87 ± 0.82 _b_	3.59 ± 0.91 _b_	2.93 ± 0.98 _ab_	2.12 ± 0.26 _a_
1-Propanol	24.41 ± 2.75 _c_	24.92 ± 2.93 _c_	28.51 ± 3.82 _c_	29.02 ± 4.13 _c_	18.21 ± 1.99 _b_	12.56 ± 1.84 _a_
Isobutanol	17.02 ± 2.11 _b_	16.82 ± 2.31 _b_	24.16 ± 2.31 _c_	23.98 ± 2.61 _c_	21.16 ± 2.92 _bc_	7.88 ± 1.61 _a_
1-Butanol	5.44 ± 0.46 _b_	2.86 ± 0.58 _a_	7.12 ± 1.23 _b_	4.89 ± 1.56 _ab_	5.11 ± 0.82 _b_	3.21 ± 0.42 _a_
2-Methyl-butanol	46.21 ± 4.53 _d_	53.49 ± 4.87 _d_	42.17 ± 5.78 _cd_	49.21 ± 6.12 _d_	32.07 ± 6.82 _c_	22.16 ± 1.93 _a_
3-Methyl-butanol	29.23 ± 2.54 _c_	31.56 ± 2.77 _c_	27.76 ± 2.88 _c_	30.11 ± 3.15 _c_	19.12 ± 2.63 _b_	14.83 ± 1.52 _a_
Isobutyl acetate	n.d.	n.d.	n.d.	n.d.	n.d.	n.d.
Ethyl butyrate	n.d.	n.d.	n.d.	n.d.	n.d.	n.d.
Hexanol	2.17 ± 0.18 _b_	1.86 ± 0.21 _b_	2.21 ± 0.32 _b_	2.02 ± 0.38 _b_	1.89 ± 0.39 _b_	1.21 ± 0.14 _a_
2-Phenyl-ethanol	30.12 ± 1.88 _b_	32.42 ± 2.59 _b_	27.23 ± 2.64 _b_	28.84 ± 3.56 _b_	25.23 ± 3.06 _ab_	22.35 ± 2.23 _a_
2-Phenyl ethyl acetate	6.85 ± 0.36 _b_	7.13 ± 0.48 _b_	6.41 ± 0.51 _b_	6.66 ± 0.62 _b_	6.02 ± 0.63 _b_	5.21 ± 0.22 _a_

Results are the mean ± SD of three replicates. Means in the same row with the same letter are not significantly different (*p* < 0.05), n.d. = not detected.

**Table 3:** Final analysis of biogenic amines from fermentations by *Saccharomyces cerevisiae* 88 alone (SC), sequential fermentation with *Saccharomyces cerevisiae* 88 and *Lachancea thermotolerans* CONCERTO™ (LT…SC), sequential fermentation with *Schizosaccharomyces pombe* 4.5 and *Lachancea thermotolerans* CONCERTO™ (LT…SK), *Schizosaccharomyces pombe* 4.5 alone (SK), and fermentations after malolactic fermentation with *Oenococcus oeni* 217 (+MLF).

Compounds	Must	SC	SC + MLF	LT···SC	LT···SC + MLF	LT···SK	SK
Histamine (mg/L)	0.13 ± 0.01 _a_	0.12 ± 0.03 _a_	0.56 ± 0.06 _b_	0.16 ± 0.05 _a_	0.51 ± 0.08 _b_	0.13 ± 0.06 _a_	0.18 ± 0.04 _a_
Tiramine (mg/L)	0.09 ± 0.01 _a_	0.08 ± 0.02 _a_	0.12 ± 0.05 _a_	0.10 ± 0.06 _a_	0.14 ± 0.09 _a_	0.11 ± 0.06 _a_	0.09 ± 0.02 _a_
Phenylethylamine (g/L)	n.d.	n.d.	n.d.	n.d.	n.d.	n.d.	n.d.
Putrescine (g/L)	0.18 ± 0.02 _a_	0.21 ± 0.03 _a_	0.25 ± 0.09 _a_	0.19 ± 0.06 _a_	0.22 ± 0.08 _a_	0.18 ± 0.07 _a_	0.16 ± 0.03 _a_
Cadaverine (g/L)	0.27 ± 0.02 _a_	0.31 ± 0.03 _a_	0.35 ± 0.07 _a_	0.29 ± 0.07 _a_	0.32 ± 0.09 _a_	0.27 ± 0.06 _a_	0.25 ± 0.03 _a_

Results represent the mean ± SD for three replicates. Means in the same row with the same letter are not significantly different (*p* < 0.05), n.d. = not detected.

**Table 4:** Final analysis of amino acids from fermentations by *Saccharomyces cerevisiae* 88 alone (SC), sequential fermentation with *Saccharomyces cerevisiae* 88 and *Lachancea thermotolerans* CONCERTO™ (LT…SC), sequential fermentation with *Schizosaccharomyces pombe* 4.5 and *Lachancea thermotolerans* CONCERTO™ (LT…SK), *Schizosaccharomyces pombe* 4.5 alone (SK), and fermentations after malolactic fermentation with *Oenococcus oeni* 217 (+MLF).

Compounds (mg/L)	SC	SC + MLF	LT…SC	LT…SC + MLF	LT…SK	SK
Aspartic acid	10.08 ± 0.61 _a_	11.56 ± 0.92 _a_	9.42 ± 1.02 _a_	10.64 ± 1.33 _a_	11.62 ± 1.78 _a_	13.76 ± 0.71 _b_
Asparagine	14.23 ± 1.08 _ab_	15.39 ± 1.32 _b_	12.48 ± 1.33 _a_	13.62 ± 1.64 _ab_	16.55 ± 1.52 _b_	23.42 ± 1.43 _c_
Serine	2.57 ± 0.41 _a_	3.71 ± 0.52 _b_	2.47 ± 0.50 _a_	3.62 ± 0.58 _b_	3.88 ± 0.64 _b_	5.11 ± 0.72 _c_
Histidine	58.42 ± 2.79 _b_	50.63 ± 3.08 _a_	61.31 ± 3.92 _b_	52.53 ± 4.21 _a_	62.93 ± 4.32 _b_	79.21 ± 3.22 _c_
Glycine	25.22 ± 1.35 _b_	29.86 ± 1.61 _c_	16.42 ± 2.16 _a_	19.76 ± 2.52 _a_	16.96 ± 2.22 _a_	26.14 ± 1.44 _b_
Arginine	56.42 ± 2.79 _b_	45.63 ± 3.08 _a_	57.31 ± 3.92 _b_	48.53 ± 3.21 _a_	55.26 ± 3.43 _b_	70.36 ± 4.79 _c_
Threonine	19.36 ± 1.89 _a_	21.26 ± 2.14 _a_	18.24 ± 2.11 _a_	20.13 ± 2.45 _a_	28.42 ± 2.93 _b_	46.58 ± 4.01 _c_
Alanine	26.31 ± 2.41 _a_	35.52 ± 2.96 _a_	25.77 ± 2.88 _a_	33.66 ± 3.17 _a_	26.52 ± 2.72 _a_	24.14 ± 1.99 _a_
Tyrosine	3.75 ± 0.21 _a_	3.89 ± 0.29 _a_	3.67 ± 0.36 _a_	3.93 ± 0.45 _a_	4.12 ± 0.98 _a_	7.38 ± 0.74 _b_
Valine	1.36 ± 0.14 _b_	1.35 ± 0.58 _b_	0.32 ± 0.28 _a_	0.33 ± 0.45 _a_	1.32 ± 0.65 _b_	5.89 ± 0.44 _d_
Tryptophan	0.24 ± 0.04 _a_	0.55 ± 0.16 _b_	0.28 ± 0.07 _a_	0.61 ± 0.21 _b_	1.05 ± 0.76 _bc_	2.11 ± 0.31 _c_
Phenylalanine	2.96 ± 0.27 _a_	3.17 ± 0.38 _a_	2.77 ± 0.41 _a_	3.02 ± 0.55 _a_	4.67 ± 0.72 _b_	6.82 ± 0.55 _c_
Isoleucine	3.16 ± 0.23 _b_	2.76 ± 0.31 _a_	5.22 ± 0.41 _d_	4.28 ± 0.67 _c_	8.87 ± 0.87 _e_	14.16 ± 0.82 _f_
Leucine	3.84 ± 0.42 _b_	2.53 ± 0.51 _a_	4.92 ± 0.62 _d_	4.11 ± 0.87 _bd_	9.96 ± 1.12 _e_	18.43 ± 1.06 _f_
Ornithine	38.21 ± 2.17 _c_	44.37 ± 2.88 _d_	36.32 ± 3.21 _c_	42.16 ± 3.94 _cd_	28.33 ± 2.51 _b_	21.15 ± 1.82 _a_
Lysine	8.52 ± 0.77 _a_	9.68 ± 0.86 _a_	8.96 ± 1.35 _a_	10.58 ± 1.33 _a_	8.78 ± 1.72 _a_	15.16 ± 1.02 _b_
Methionine	1.11 ± 0.18 _a_	1.82 ± 0.31 _b_	1.08 ± 0.24 _a_	1.68 ± 0.44 _ab_	1.46 ± 0.31 _ab_	2.45 ± 0.29 _c_

Results represent the mean ± SD for three replicates. Means in the same row with the same letter are not significantly different (*p* < 0.05).

**Table 5:** Colour measurements in the wines produced by the different fermentation assays: *Saccharomyces cerevisiae* 88 alone (SC), sequential fermentation with *Saccharomyces cerevisiae* 88 and *Lachancea thermotolerans* CONCERTO™ (LT···SC), sequential fermentation with *Schizosaccharomyces pombe* 4.5 and *Lachancea thermotolerans* CONCERTO™ (LT…SK), *Schizosaccharomyces pombe* 4.5 alone (SK), and fermentations after malolactic fermentation with *Oenococcus oeni* 217 (+MLF).

Compounds	SC	SC + MLF	LT…SC	LT…SC + MLF	LT…SK	SK
420 nm	6 ± 1 _a_	6 ± 1 _a_	7 ± 1 _a_	7 ± 1 _a_	7 ± 1 _a_	08 ± 1 _a_
520 nm	8 ± 1 _b_	6 ± 1 _a_	9 ± 1 _b_	7 ± 1 _a_	9 ± 1 _bc_	10 ± 1 _c_
620 nm	2 ± 1 _a_	2 ± 1 _a_	1 ± 1 _a_	1 ± 1 _a_	02 ± 1 _a_	2 ± 1 _a_
Cia<	16 ± 1 _ab_	14 ± 1 _a_	17 ± 1 _ab_	15 ± 1 _a_	18 ± 1 _bc_	20 ± 1 _c_
Hue	0.75 ± 0.02 _a_	1.00 ± 0.02 _b_	0.77 ± 2 _a_	1.00 ± 0.02 _b_	0.77 ± 0.02 _a_	0.80 ± 0.02 _a_

Results are the mean ± SD of three replicates. Means in the same row with the same letter are not significantly different (*p* < 0.05).
